# Low fluid shear stress stimulates the uptake of noxious endothelial extracellular vesicles via MCAM and PECAM‐1 cell adhesion molecules

**DOI:** 10.1002/jev2.12414

**Published:** 2024-10-14

**Authors:** Pierre‐Michaël Coly, Shruti Chatterjee, Fariza Mezine, Christelle El Jekmek, Cécile Devue, Thomas Nipoti, Stephane Mazlan, Maribel Lara Corona, Florent Dingli, Damarys Loew, Guillaume van Niel, Xavier Loyer, Chantal M. Boulanger

**Affiliations:** ^1^ Université Paris‐Cité, PARCC INSERM Paris France; ^2^ Institute of Psychiatry and Neuroscience of Paris (IPNP), Université de Paris, INSERM Paris France; ^3^ Institut Curie, PSL Research University, Centre de Recherche, CurieCoreTech Spectrométrie de Masse Protéomique Paris France; ^4^ GHU Paris Psychiatrie et Neurosciences, Hôpital Sainte Anne Paris France

**Keywords:** endothelial cells, extracellular vesicles, MCAM, mitochondria, PECAM1, shear stress, uptake

## Abstract

Atherosclerotic lesions mainly form in arterial areas exposed to low shear stress (LSS), where endothelial cells express a senescent and inflammatory phenotype. Conversely, areas exposed to high shear stress (HSS) are protected from plaque development. Endothelial extracellular vesicles (EVs) have been shown to regulate inflammation and senescence, and therefore play a crucial role in vascular homeostasis. Whilst previous studies have shown links between hemodynamic forces and EV release, the effects of shear stress on the release and uptake of endothelial EVs remains elusive. We aim to decipher the interplay between these processes in endothelial cells exposed to atheroprone or atheroprotective shear stress. Confluent HUVECs were exposed to LSS or HSS for 24 h. Large and small EVs were isolated from conditioned medium by centrifugation and size exclusion chromatography. They were characterised by TEM, Western blot, tunable resistive pulse sensing, flow cytometry and proteomics. Uptake experiments were performed using fluorescently‐labelled EVs and differences between groups were assessed by flow cytometry and confocal microscopy. We found that levels of large and small EVs in conditioned media were fifty and five times higher in HSS than in LSS conditions, respectively. In vivo and in vitro uptake experiments revealed greater EV incorporation by cells exposed to LSS conditions. Additionally, endothelial LSS‐EVs have a greater affinity for HUVECs than HSS‐EVs or EVs derived from platelets, erythrocytes and leukocytes. Proteomic analysis revealed that LSS‐EVs were enriched in adhesion proteins (PECAM1, MCAM), participating in EV uptake by endothelial cells. LSS‐EVs also carried mitochondrial material, which may be implicated in elevating ROS levels in recipient cells. These findings suggest that shear stress influences EV biogenesis and uptake. Given the major role of EVs and shear stress in vascular health, deciphering the relation between these processes may yield innovative strategies for the early detection and treatment of endothelial dysfunction.

## 1 INTRODUCTION

Hemodynamic forces are a major determinant of atherosclerotic plaque formation. Shear stress, the mechanical force exerted by the blood flow on the vascular wall, has a profound impact on the phenotype of endothelial cells (Chiu & Chien, 2011; Gimbrone & García‐Cardeña, 2013; Hahn & Schwartz, 2009). Plaques preferentially form in regions where the blood flow is turbulent and oscillatory, such as arterial bifurcations or curvatures. Here, endothelial cells are exposed to low shear stress (LSS), which favour apoptosis, senescence and inflammation. Conversely, regions of laminar blood flow are exposed to high shear stress (HSS) and are relatively protected from atherosclerotic lesions (Davies et al., 2013; Tricot et al., 2000; Warboys et al., 2014). Among the affected pathways, our group and others demonstrated that LSS impacts the endothelial endolysosomal system, namely through reduced lysosomal acidity and perturbed autophagic flux (Chung et al., 2022; Liu et al., 2015; Vion et al., 2017).

Extracellular vesicles (EVs) are submicron membrane particles released by all cell types via diverse mechanisms. The two main EV subpopulations are exosomes (50–150 nm) and microvesicles (100–1000 nm). The former is created by inward budding of the endosomal membrane, thereby forming intraluminal vesicles, and secreted when multivesicular endosomes fuse with the plasma membrane. Microvesicles, on the other hand, are generated by outward budding of the plasma membrane (van Niel et al., 2018). Long considered as only a means of eliminating cellular waste, EVs are now believed to be key players in cellular communication, as they can carry biological information (proteins, lipids, nucleic acids) between cells (van Niel et al., 2022). Being in direct contact with the blood flow means that endothelial cells can easily release EVs into the circulation, this makes them the second largest contributor of circulating EVs behind platelets (Mathiesen et al., 2021). The endothelium also interacts with circulating EVs, as they have been seen rolling, arresting and accumulating on the endothelial cell wall in vivo (Hyenne et al., 2019; Verweij et al., 2019). The composition and subsequent effect of EVs seems to be largely dependent on the state of the parental cell. In that sense, EVs are involved in several physiopathological processes, many of which underlie the progression of atherosclerosis. EVs derived from injured of inflamed endothelial cells have been shown to carry specific microRNAs, cytokines or adhesion proteins, and have been implicated in monocyte activation, endothelial permeability and smooth muscle cell proliferation (Coly & Boulanger, 2022). Whilst it is known that shear stress is a major determinant of EV production (Vion et al., 2013), we have relatively little data on the impact of these forces on the full composition of endothelial EVs. Similarly, we know little on how the level of shear stress affects EV endocytosis by endothelial cells, and the consequences on the endothelial phenotype. Interestingly, recent data have shown that the process of EV release can intersect with autophagy, be it by the formation of secretory amphisomes upon fusion of autophagosomes with multivesicular endosomes, or the fusion of mature autophagosomes with the plasma membrane (van Niel et al., 2022). These mechanisms expand the variety of EV subpopulations and result in the release of heterogenous content into the extracellular space, including digestion material.

By impacting the endolysosomal network we hypothesise that shear stress can alter the endocytosis of EVs by endothelial cells, as well as the composition of secreted EVs. In this study, we found that endothelial cells exposed to LSS, and therefore prone to developing atherosclerotic plaques incorporated more EVs than cells exposed to HSS via macropinocytosis and a clathrin‐dependent pathway. Endothelial cells exposed to LSS released EVs that were enriched in adhesion and mitochondrial proteins. Functionally, this resulted in LSS‐EVs being more readily endocytosed than HSS‐EVs, as well as having a pro‐oxidative effect on recipient cells.

## 2 MATERIALS AND METHODS

### 2.1 Cell culture

Human Umbilical Vein Endothelial Cells (HUVEC; passage 2–4; 20 different primary cultures; PromoCell) were cultured on 0,2% gelatin‐coated slides (18.75 cm^2^), in Endothelial Cell Basal Medium (ECBM, PromoCell), supplemented with 2% Foetal Calf Serum (PromoCell), growth factors (0.4% ECGS, 0.1 ng/mL EGF, 1 ng/mL ß‐FGF), heparin (90 μg/mL), hydrocortisone (1 μg/mL), Amphotericin B (10 μg/L, Gibco), Streptomycin (100 IU/mL, Gibco) and Penicillin (100 IU/mL, Gibco). Murine endothelial cells, SVEC4‐10 (ATCC), were cultured in DMEM supplemented with Foetal Calf Serum (10%), Amphotericin B (10 μg/L), Streptomycin (100 IU/mL) and Penicillin (100 IU/mL, Gibco). The human monocytic cell line, THP‐1 (ATCC), was maintained in RPMI 1640 medium supplemented with Foetal Calf Serum (10%), Amphotericin B (10 μg/L), Streptomycin (100 IU/mL) and Penicillin (100 IU/mL, Gibco).

### 2.2 In vitro shear stress system

A controlled level of laminar shear stress was applied to confluent endothelial cells using a parallel plate chamber connected to a perfusion circuit driven by a peristaltic pump (Gilson). Before use, the foetal calf serum was ultracentrifuged (100,000 × *g*, 90 min) and the complete medium was filtered on a 0.1 μm membrane to remove serum EVs. Confluent cells (≈ 1 million per slide) were placed in the perfusion system for 24 h, in a sterile 5% CO_2_ incubator at 37°C. LSS (2 dyn/cm^2^) or HSS (20 dyn/cm^2^ for HUVECs and 40 dyn/cm^2^ for SVEC4‐10) was calculated using Poiseuille's law.

### 2.3 EV isolation

Following exposure to shear stress, the conditioned media (20 mL per slide) were collected and first centrifuged at 600 × *g* for 15 min at 4°C to remove cell debris, then at 4500 × *g* for 20 min at 4°C to remove apoptotic bodies. The resulting supernatants were subjected to differential centrifugations at 20,500 × *g* for 120 min to pellet lEVs and 100,000 × *g* for 90 min to pellet sEVs. EVs were resuspended in filtered PBS, purified by size exclusion chromatography (qEVsingle/70 nm, Izon, New Zealand), and stored at −80°C until use.

Blood was collected in citrated tubes. Tubes were centrifuged at 2500 × *g* for 15 min. The platelet‐free plasma supernatant (PFP) was collected and subjected to size exclusion chromatography to isolate EVs. Blood cells (platelets, neutrophils, peripheral blood mononuclear cells and red blood cells) were separated on a density gradient (Granulosep, Eurobio) via a 30 min centrifugation at 700 × *g*. EVs were obtained by incubating red blood cell with CaCl (5 mM) and other cell types with the calcium ionophore A23187 (1 mM, C9275, Sigma) for 30 min at 37°C. Samples were then centrifuged at 600 × *g* for 10 min and 4500 × *g* for 20 min to eliminate cells and debris. lEVs were obtained as described above.

### 2.4 Transmission electron microscopy

EVs were directly deposited on formvar/carbon coated copper/palladium grids for 20 min at room temperature and fixed with PFA 2%/0.1 M phosphate buffer (Corona et al., 2023). Negative staining was performed using uranyl acetate 0.4% in methylcellulose. The samples were analysed with a 120 kV transmission electron microscope (Tecnai Spirit G2; ThermoFischer Scientific) equipped with a 4 k CCD camera (On view 4 k × 4 k Gatan).

### 2.5 Tunable resistive pulse sensing measurements

EVs were quantified using NP400 (lEVs) and NP150 (sEVs) nanopores (Izon, New Zealand). The measurements were calibrated with polystyrene beads CPC 400 (350 nm mode diameter) and CPC 200 (210 nm mode diameter) respectively. Concentrations were expressed as EVs normalised to the volume of conditioned medium collected after exposure to shear stress.

### 2.6 EV flow cytometry

lEVs were incubated with the membrane probe MemBright (1 μM, Idylle Labs) in 50 μL of PBS for 30 min at room temperature in the dark. They were then rinsed in 1 mL of PBS and pelleted by centrifugation (20,500 × *g*). EVs were resuspended in PBS and used immediately for flow cytometry experiments. lEVs were analysed on a Cytoflex flow cytometer (Beckman Coulter, USA) using Megamix‐Plus SSC beads and Megamix‐Plus SSC beads (Biocytex, France) to define events of 0.1–0.9 μm diameter size. For experiments using fluorescence‐conjugated antibodies to stain lEV surface markers, antibodies were first centrifuged for 5 min at 13,000 × *g* at 4°C before being added to EV samples. lEVs were defined as events stained by MemBright. Antibodies were as follows: PECAM1 (IM2409, Beckman Coulter), MCAM (A07483, Beckman Coulter), α5 integrin (563578, BD Biosciences), α6 integrin (561894, BD Biosciences) and β1 integrin (130‐101‐271, Miltenyi Biotec). Corresponding isotype controls were as follows: IgG1 Mouse‐PE (A0779, Beckman Coulter), IgG2a Mouse‐PE (A09142, Beckman Coulter), IgG1κ Mouse‐AF647 (565571 BD Biosciences), IgG2a κ Rat‐PE (551799, BD Biosciences) and IgG1κ Mouse‐APC (130‐113‐196, Miltenyi Biotec), respectively. To assess levels of mitochondrial and lysosomal material, EVs were stained with Mitotracker Deep Red (400 nM, ThermoFisher Scientific) and LysoTracker Deep Red (50 nM, ThermoFisher Scientific) respectively. Controls include detergent lysis, buffer‐only, buffer with reagent (without EVs) and unstained samples.

### 2.7 Immunoblotting

HUVECs were washed with cold PBS and lysed in Radioimmunoprecipitation assay (RIPA) buffer. For EV samples, RIPA (2X) was added to equal volume of EVs suspended in PBS. Protein content was quantified using the Micro BCA Protein Assay kit (ThermoFisher Scientific). Lysates were mixed with the reducing sample buffer for electrophoresis and subsequently transferred onto nitrocellulose membranes (Bio‐Rad). Equal loading was checked using Ponceau Red solution. Membranes were incubated with primary antibodies overnight at 4°C, with constant agitation. After secondary antibody incubation (1 h, room temperature), immunodetection was performed using Clarity Western ECL Substrate, and the chemiluminescent signal was revealed using the LAS‐4000 imaging system and quantified with MultiGauge software (Fujifilm, Japan). Antibodies were as follows: CD9 (Merck Millipore, CBL162), CD63 (MBL Life Science, D263‐3), HSC70 (Enzo Life Sciences, SPA815), TOMM20 (Abcam, ab46798), LAMP1 (BD Biosciences 611043), GAPDH (Merk Millipore, mab‐374), α6 integrin (Cell Signalling Technology, 3750), β1 integrin (Abcam, ab52971), MCAM long and short (generously provided by Dr Aurélie Leroyer, Aix‐Marseille University), HRP‐linked Anti‐rabbit (GE Healthcare, NA934), HRP‐linked Anti‐mouse (GE Healthcare, NXA931), HRP‐linked Anti‐rat (Jackson Immunity, 112‐035‐150).

### 2.8 Uptake assay by flow cytometry

lEVs and sEVs were labelled with the fluorescent membrane probe MemBright (1 μM) in 50 μL of PBS for 30 min at room temperature in the dark. They were then rinsed in 1 mL of PBS and pelleted by centrifugation (20,500 × *g* and 100,000 × *g* for lEVs and sEVs, respectively). EVs were resuspended in PBS and stored at 4°C (>24 h) until use. Following shear stress experiments, cells were washed with warm media and immediately incubated, for indicated periods, with labelled EVs (7.5 million/mL). Cells were then washed with PBS, detached with Trypsin and the fluorescent signal intensity per cell was measured by flow cytometry. Cell viability was assessed using the Fixable Viability Dye eFluor 780 (ThermoFisher Scientific). When indicated, cells were treated with amiloride (1 mM, Sigma, A7410), nystatin (50 μM, Sigma N1638) or chlorpromazine (20 μM, Sigma, C8138) 1 h before incubation with EVs. For surface protein neutralization, EVs were pre‐incubated with blocking antibodies for 30 min at room temperature, then centrifuged to eliminate unbound antibodies. EVs were then resuspended in warm media and deposited on cells. Blocking antibodies were as follows: PECAM1 (MA3100, ThermoFisher Scientific), MCAM (clone S‐Endo 1, BioCytex), β1 integrin (Biolegend, 921303), α5 integrin (Biolegend, 921704), α6 integrin (Biolegend, 313602).

### 2.9 Immunofluorescent staining and microscopy in vitro

After shear stress, HUVECs were fixed with 4% PFA and blocked with 5% BSA in PBS. Cells were incubated with primary antibody overnight at 4°C (Cadherin‐5, Santa Cruz Biotechnology, sc‐6458). And then with AlexaFluor‐conjugated secondary antibody (ThermoFisher Scientific). Nuclei were stained with DAPI (0.1 μg/mL).

### 2.10 ROS measurement

To assess ROS levels following exposure to EVs, we used the CellROX Deep Red Reagent (Thermo Fisher Scientific, C10422), a fluorogenic probe designed to reliably measure ROS inside living cells. This cell‐permeable dye is in a non‐fluorescent reduced state whilst outside the cell and exhibits excitation/emission maxima at 640/665 nm upon oxidation. HUVECs were incubated with 5 μM CellROX for 30 min at 37°C. Cells were the then fixed and imaged by confocal microscopy.

### 2.11 In vivo biodistribution studies

Mice used in the study were of C57BL/6 genetic background. Membright‐labelled SVEC4‐10‐derived EVs (500 × 10^6^ in 150 μL PBS) were injected in the retro‐orbital sinus. PBS vehicle and EV‐labelling solution supernatant was used as controls. Mice were sacrificed 30 min post‐injection and their aortas were injected in situ with PBS supplemented with 4% PFA. After dissection, the aortas were blocked with 5% BSA for 20 min and stained with anti‐Cadherin‐5 antibody (Santa Cruz biotechnology, sc‐6458) and DAPI. For all mice, 10 confocal images were obtained from regions in the aortic arch (LSS) and the thoracic aorta (HSS). All experiments were performed in accordance with the European Community guidelines for the care and use of laboratory animals (no. 07430) and were approved by the institutional ethical committee (no. 02526.02).

### 2.12 Proteomics and mass spectrometry analysis

#### 2.12.1 Sample preparation

After isolation, EVs were pelleted and lysed in a solution of urea (8 M) and ammonium bicarbonate (200 mM). Protein content was quantified using the Micro BCA Protein Assay kit (ThermoFisher Scientific). A total of 10 μg of each sample in 10 μL of 8 M urea, 200 mM ammonium bicarbonate were reduced in 5 mM dithiothreitol, pH 8 by vortexing at 57°C for 30 min. After cooling to room temperature, cysteines were alkylated by adding 10 mM iodoacetamide for 30 min in the dark. After diluting to 1 M urea with 200 mM ammonium bicarbonate pH 8.0, samples were digested by adding first 0.4 μg trypsine/LysC (Promega) during 2 h and then 0.4 μg overnight, by vortexing at 37°. Samples were then loaded onto homemade C18 StageTips for desalting. Peptides were eluted using 40/60 MeCN/H2O + 0.1% formic acid, vacuum concentrated to dryness and reconstituted in 10 μL injection buffer (0.3% TFA) before nano‐LC‐MS/MS analysis.

#### 2.12.2 LC‐MS/MS analysis

Online chromatography was performed using an RSLCnano system (Ultimate 3000, Thermo Fisher Scientific) coupled to an Orbitrap Fusion Tribrid mass spectrometer (Thermo Fisher Scientific). Peptides were trapped in a C18 column (75 μm inner diameter × 2 cm; nanoViper Acclaim PepMap 100, Thermo Fisher Scientific) with buffer A (2:98 MeCN:H2O in 0.1% formic acid) at a flow rate of 4.0 μL/min over 4 min. Separation was performed on a 50 cm × 75 μm C18 column (nanoViper Acclaim PepMapTM RSLC, 2 μm, 100Å, Thermo Scientific), regulated to a temperature of 55 °C with a linear gradient of 5% to 25% buffer B (100% MeCN in 0.1% formic acid) at a flow rate of 300 nL/min over 100 min. Full‐scan MS was acquired using an Orbitrap Analyzer with the resolution set to 120,000, and ions from each full scan were higher‐energy C‐trap dissociation (HCD) fragmented and analysed in the linear ion trap.

#### 2.12.3 Data analysis

For identification, the data were searched against the Homo Sapiens UP000005640 UniProt database using Sequest HT through Proteome Discoverer (v.2.2). Enzyme specificity was set to trypsin and a maximum of two missed cleavages sites were allowed. Oxidised methionine, N‐terminal acetylation and carbamidomethylation of cysteines were set as variable modifications. Maximum allowed mass deviation was set to 10 ppm for monoisotopic precursor ions and 0.6 Da for MS/MS peaks. The resulting files were further processed using myProMS (https://github.com/bioinfo‐pf‐curie/myproms; Poullet et al., 2007) v.3.9.2 (work in progress). False‐discovery rate (FDR) was calculated using Percolato (The et al., 2016) and was set to 1% at the peptide level for the whole study. Label‐free quantification was performed using peptide extracted ion chromatograms (XICs), computed with MassChroQ (Valot et al., 2011) v.2.2.1. For protein quantification, XICs from proteotypic peptides shared between compared conditions (TopN matching) were used. Missed cleavages were allowed for quantification. Median and scale normalization at peptide level was applied on the total signal to correct the XICs for each biological replicate (*N* = 5). To estimate the significance of the change in protein abundance, a linear model (adjusted on peptides and biological replicates) was performed, and *p*‐values were adjusted using the Benjamini–Hochberg FDR procedure.

The mass spectrometry proteomics raw data have been deposited to the ProteomeXchange Consortium via the PRIDE (Perez‐Riverol et al., 2022) partner repository dataset identifier. Project accession: PXD041453.

### 2.13 Statistical analysis

Data are expressed as mean ± SEM for all experiments. Statistical analyses were performed using the GraphPad Prism 9 statistical package. Normality of the data were confirmed using Shapiro‐Wilk test and accordingly different statistical tests were used as described in legends. For data that follow gaussian distribution, *t‐*test or one‐way Anova were used. For data that do not follow gaussian distribution, Wilcoxon or Friedman tests were used. *p*‐values smaller than 0.05 were considered as statistically significant. *, *p* < 0.05, **, *p* < 0.01, ***, *p* < 0.001, ****, *p* < 0.0001.

## 3 RESULTS

### 3.1 Shear stress affects the concentration of EVs found in endothelial cell conditioned media

**FIGURE 1:**
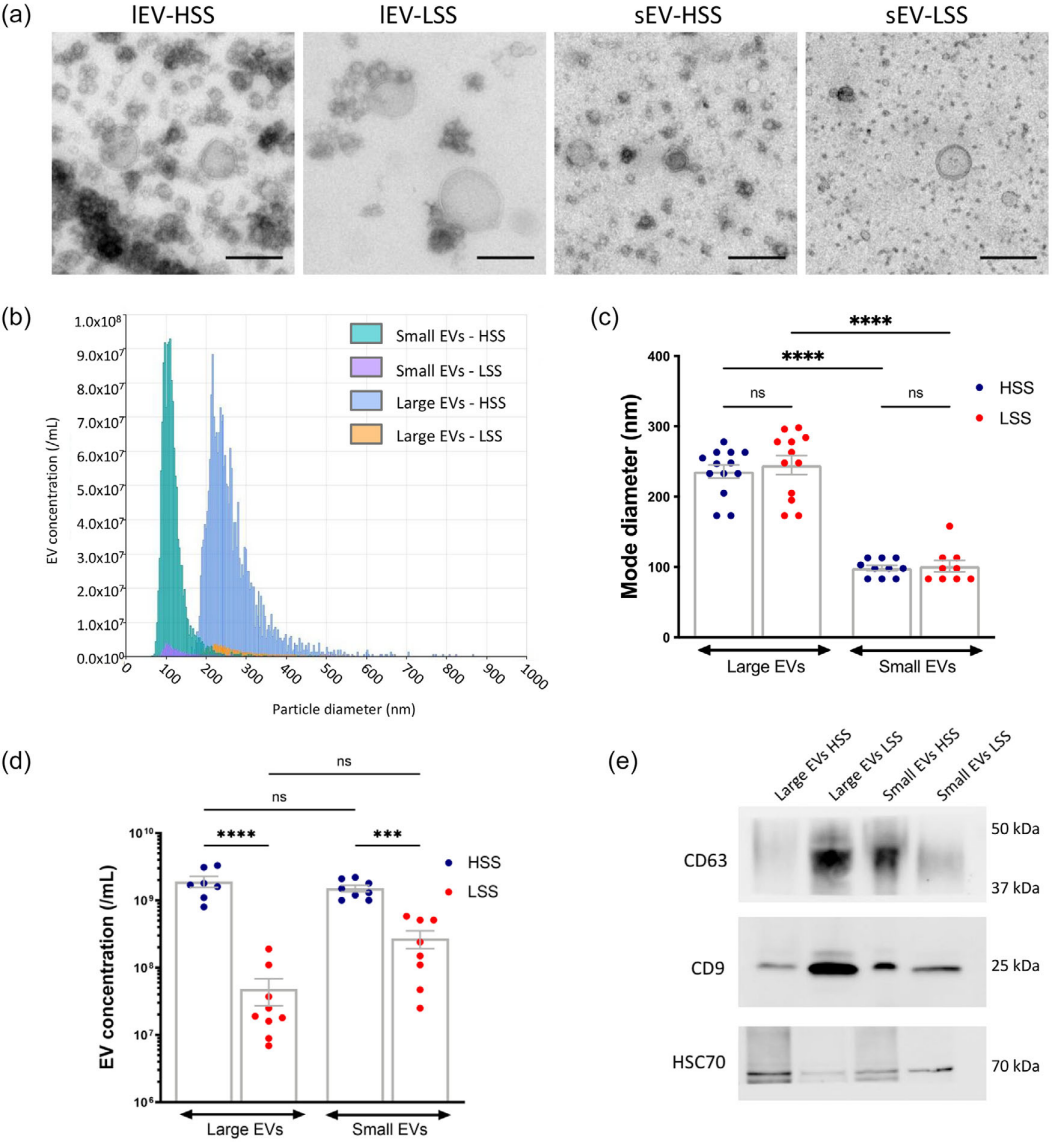
Characterization of endothelial EVs produced under HSS or LSS conditions. (a) HUVECs were exposed to HSS or LSS for 24 h. Large (lEVs) and small (sEVs) EVs were isolated by differential centrifugation and size exclusion chromatography and observed by transmission electron microscopy. Scale bar = 200 nm. (b) Representative size distribution analysis by tunable resistive pulse sensing (TRPS). (c) EV mode diameter determined by TRPS. (d) EV concentration determined by TRPS. (e) Representative Western blot for CD63, CD9 and HSC70 in lEVs and sEVs produced under HSS or LSS conditions. Data represent means ± SEM of 7–13 independent experiments. ***p < 0.001, ****p < 0.0001; One‐way Anova.

HUVECs were exposed to HSS or LSS conditions for 24 h. Large (lEVs) and small (sEVs) EVs were then isolated from the conditioned media by differential centrifugation followed by size exclusion chromatography, and their morphology was observed by transmission electron microscopy (Figure 1a). Quantification by tunable resistive pulse sensing revealed that sEVs and lEVs had a mode diameter of approximately 100 nm and 230 nm respectively, with no significant difference between HSS and LSS EVs. In terms of concentration, we found substantially more EVs in the conditioned medium of cells exposed to HSS, compared to LSS (Figure 1b–d). Finally, Western blot analysis revealed that EVs harboured the transmembrane proteins CD63, CD9 and the cytosolic protein HSC70 (Figure 1e).

### 3.2 EVs produced under LSS conditions are more readily taken up than EVs produced under HSS

**FIGURE 2:**
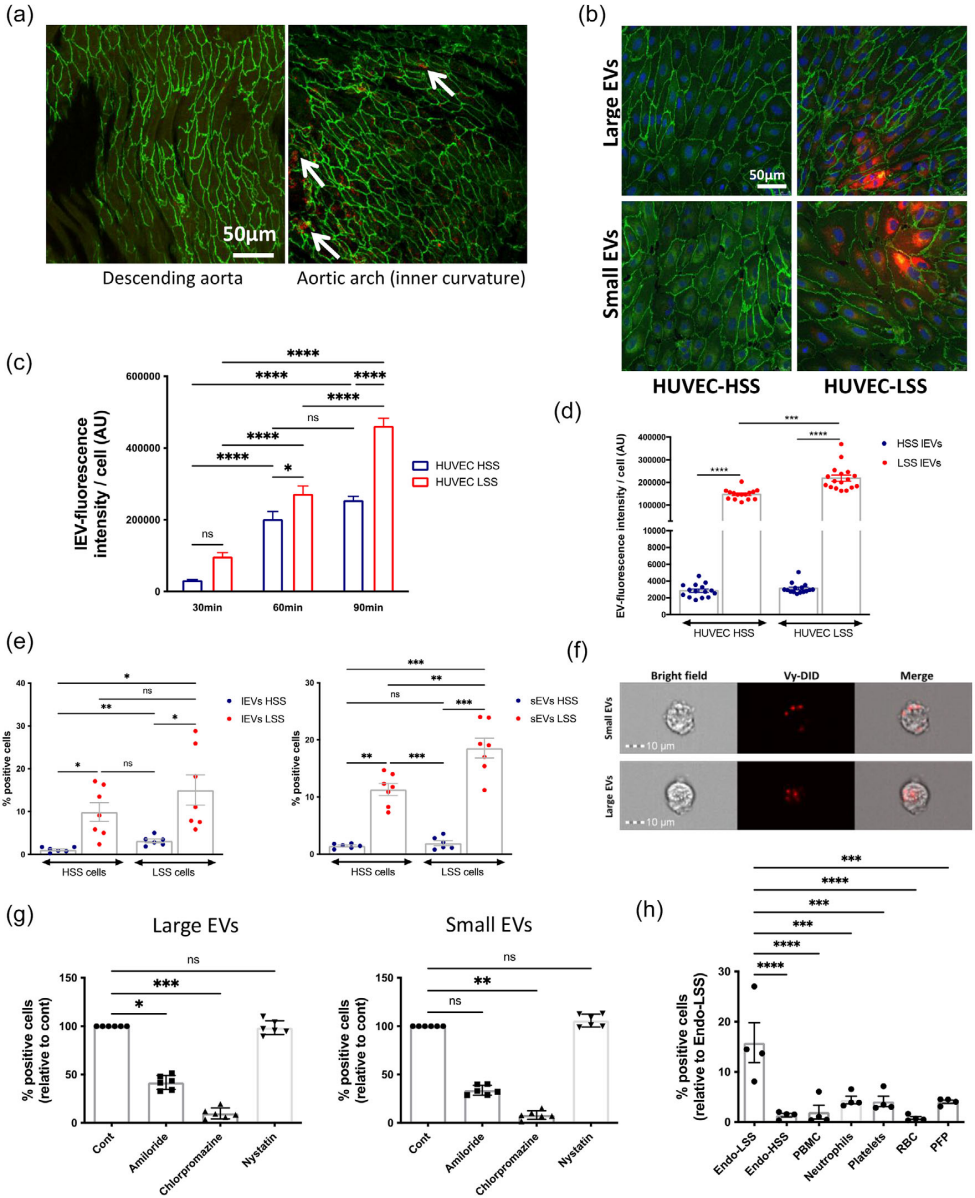
LSS‐EVs are more readily taken up than HSS‐EVs. (a) Fluorescently‐labelled SVEC4‐10 LSS‐lEVs (red) were injected into the mouse bloodstream. Animals were sacrificed after 30 min and aortas were harvested. Endothelial cells (green; Cadherin‐5 staining) were imaged by confocal microscopy. White arrows indicate regions of EV accumulation. (b) Fluorescently‐labelled HUVEC LSS‐lEVs (red) were incubated for 90 min with HUVECs exposed to HSS or LSS conditions for 24 h. Cells were fixed, stained for Cadherin‐5 (green) and imaged by confocal microscopy. (c) Fluorescently‐labelled HUVEC‐derived LSS‐lEVs were incubated for varying periods min with HUVECs exposed to HSS (blue) or LSS (red) conditions for 24 h. Representative quantifications of EV signal per cell from confocal images captured at different time points (15 fields per condition, *p < 0.05, ****p < 0.0001, One‐way Anova). (d) Fluorescently‐labelled HUVEC‐derived HSS‐lEVs (blue) or LSS‐lEVs (red) were incubated for 90 min with HUVECs exposed to HSS or LSS conditions for 24 h. Representative quantifications of EV signal per cell from confocal images (15–17 fields per condition, ***p < 0.001, ****p < 0.0001, One‐way Anova). (e) Fluorescently‐labelled HUVEC‐derived HSS‐ (blue) or LSS‐ (red) lEVs (left) or sEVs (right) were incubated for 90 min with HUVECs exposed to HSS or LSS conditions for 24 h. % of cells positive for EV signal was analysed by flow cytometry. Data represent means ± SEM of seven independent experiments, *p < 0.05, **p < 0.01, ***p < 0.001, One‐way Anova. (f) Fluorescently‐labelled HUVEC‐derived LSS lEVs or sEVs were incubated with HUVECs exposed to LSS conditions for 24 h. Cells were and visualised by image flow cytometry. (g) Fluorescently‐labelled HUVEC‐derived LSS lEVs (left) or sEVs (right) were incubated for 90 min, in the presence of endocytosis inhibitors, with HUVECs exposed to LSS conditions for 24 h. % of cells positive for EV signal, relative to Cont, was analysed by flow cytometry. Data represent means ± SEM, relative to Cont, of six independent experiments, *p < 0.05, **p < 0.01, *** *p* < 0.001, Friedman test. (h) Fluorescently‐labelled lEVs derived from HUVECs exposed to LSS (Endo‐LSS) or HSS (Endo‐LSS), peripheral blood mononuclear cells (PMBC), neutrophils, platelets, red blood cells (RBC) or platelet free plasma (PFP) were incubated for 90 min with HUVECs exposed to LSS conditions for 24 h. % of cells positive for EV signal was analysed by flow cytometry. Data represent means ± SEM of four independent experiments, ***p < 0.001, ****p < 0.0001, One‐way Anova.

To assess if endothelial EVs could be taken up by endothelial cells in vivo, we labelled murine endothelial cell‐derived EVs (SVEV4‐10) with the fluorescent lipophilic dye Membright as described in Hyenne et al. (2019). Mice were injected with labelled‐EVs and sacrificed after 30 min to assess EV incorporation into vascular endothelial cells. Confocal imaging of mouse aortas revealed that HSS‐lEVs had little retention in endothelial cells, as endothelial fluorescence was below detection levels. Conversely LSS‐lEVs were visible in the mouse aorta following injection and seemed to preferentially accumulate in the inner aortic arch (region of LSS) when compared to the thoracic aorta (region of HSS; Figure 2a and Figure S1). We then performed in vitro EV‐uptake assays using fluorescently‐labelled endothelial EVs produced under HSS or LSS conditions. We deposited these EVs on HUVECs that had been exposed to HSS or LSS for 24 h, and assessed the rate of EV accumulation by confocal microscopy and flow cytometry. Confocal imaging revealed that HUVECs exposed to LSS took up more fluorescently‐labelled EVs than HUVECs exposed to HSS (Figure 2b,c), thus corroborating our in vivo observations (Figure 2a). Here also, we found that LSS‐lEVs were more readily detectable in endothelial cells, suggesting greater uptake of these vesicles compared to HSS‐lEVs (Figure 2d). sEV uptake was below detection levels by this method. Similar data were obtained when analysing cells by flow cytometry. For these experiments, cells viability was assessed using the eFluor 780 dye, to ensure levels of viability >95%. There was no difference in viability between cells exposed to HSS or LSS (Figure S3). LSS‐EVs were detectable in 10%–20% of cells, whilst HSS‐EVs were detectable in less than 5% of cells (Figure 2e). Further examination by imaging flow cytometry showed that the fluorescent EV pattern often appeared to be located within the cytoplasm, rather than only at the cell surface, which suggests that these EVs are being endocytosed by HUVECs (Figure 2f). To ensure that this was the case, we targeted several endocytic pathways previously described for EV uptake, that is, macropinocytosis, clathrin‐mediated endocytosis and caveolin‐mediated endocytosis using pharmacological inhibitors: amiloride, chlorpromazine and nystatin respectively. We found that perturbing macropinocytosis and clathrin‐mediated endocytosis reduced the EV signal detected in cells, which indicates that these pathways are necessary for endothelial EV uptake by HUVECs (Figure 2g). We next sought to compare the uptake levels of endothelial EVs to plasma‐derived EVs, as well as EVs isolated from circulating cells: peripheral blood mononuclear cells, neutrophils, platelets and red blood cells. Whilst all EV types seemed to be taken up by HUVECs, endothelial EVs produced under LSS conditions displayed the highest rate of uptake, suggesting a higher affinity of endothelial cells for these vesicles (Figure 2h).

### 3.3 Quantitative proteomic analysis of large and small EVs produced under HSS and LSS conditions

**FIGURE 3:**
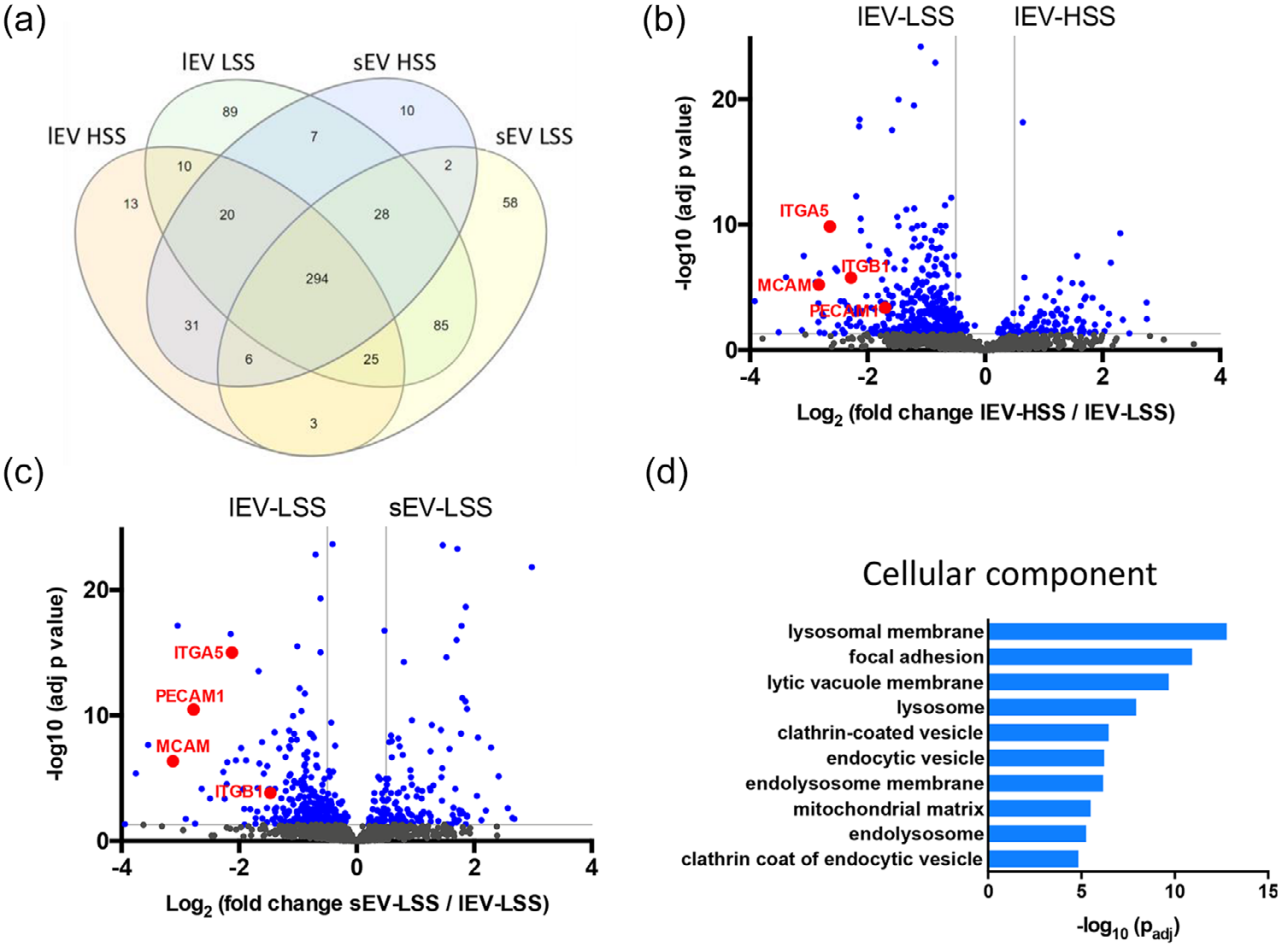
Qualitative and quantitative proteomic analysis of endothelial EVs produced under HSS and LSS. (a) Venn diagram showing the distribution of proteins qualitatively identified in each group by at least three peptides in five biological replicates. Quantitative analysis of proteins present in HSS‐lEVs (b) or LSS‐sEVs (c) compared LSS‐lEVs is shown as Volcano plots. X axis = log_2_ (fold change), Y axis = −log10 (adj *p‐*value). The horizontal grey line indicates *p‐*value = 0.05, vertical grey lines indicate absolute fold‐change = 2. (d) Gene Ontology, cellular component terms associated with proteins enriched in LSS‐lEVs.

To shed light on the mechanisms underlying the higher uptake of LSS‐EVs, we performed a proteomic analysis of large and small EVs produced under HSS and LSS conditions, from five biological replicates. We identified a variety of proteins traditionally found in mammalian EVs, including tetraspanins and heat shock proteins (Table S1). Furthermore, Gene Ontology (GO) term enrichment analysis of all identified proteins showed terms relating to extracellular vesicles (Figure S2). Qualitative analysis, evaluating the presence of proteins identified by at least three distinct peptides, indicated that most proteins were common to all four groups (Figure 3a). Each group also contained a unique set of proteins, with LSS EV sets being more substantial. GO term enrichment analysis of unique proteins revealed accumulation of cell adhesion proteins in “lEV‐LSS” and ribosome proteins in “sEV‐LSS.”

As most proteins were common between all groups, we then performed a quantitative comparison of their amounts in LSS‐lEVs versus HSS‐lEVs (Figure 3b) or LSS‐lEVs vs LSS‐sEVs (Figure 3c). GO term classification of proteins significantly (*p* < 0.05) enriched in protein comparison (adjusted *p*‐value < 0.05, fold change ≥2 and 3 peptides) in LSS‐lEVs compared to HSS‐lEVs or LSS‐sEVs, highlighted terms “extracellular space,” “cell junction,” “focal adhesion” which suggests that LSS‐lEVs are equipped with a higher range of adhesion proteins which may be essential for their uptake by endothelial cells (Figure 3d). These adhesion proteins include PECAM1, MCAM, as well as integrins α5 and β1, which were approximately 3–9 times more abundant in LSS‐lEVs. Interestingly, we found that endolysosomal and mitochondrial proteins were also more enriched in LSS‐lEVs. This may be a sign of increased secretion of digestion products. Thus, quantitative proteomic analysis demonstrated that shear stress alters the protein content of endothelial EVs.

### 3.4 LSS‐lEVs are enriched in adhesion proteins

**FIGURE 4:**
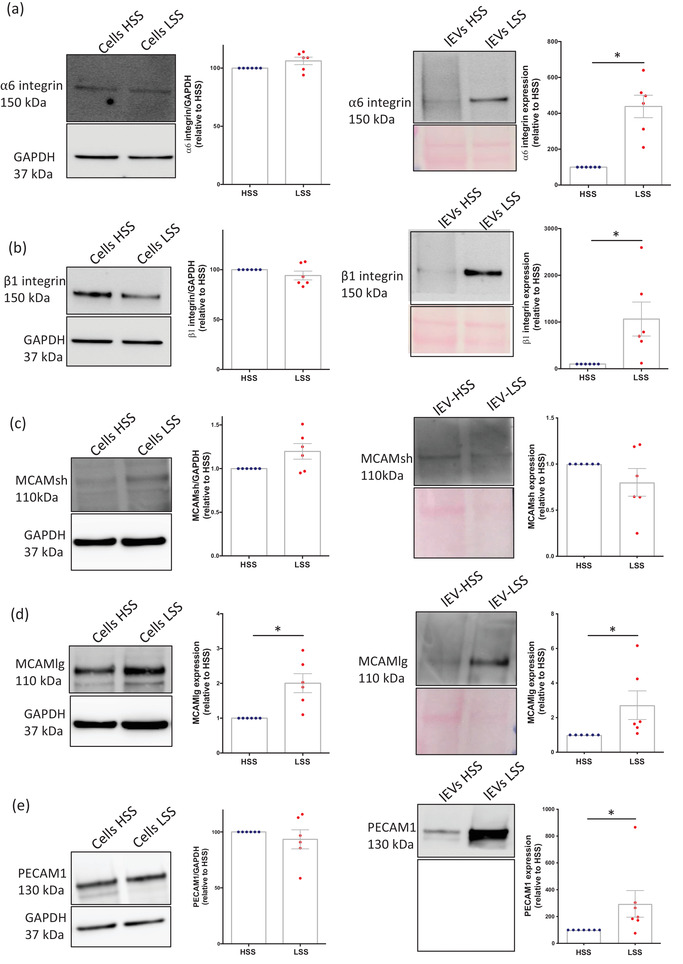
Adhesion proteins are more abundant in LSS‐lEVs. Western blot analysis of α5 integrin (a), β1 integrin (b), MCAM short isoform (c), MCAM long isoform (d) and PECAM1 (e) levels in HUVEC cellular (left panels) and lEV (right panels) protein lysates. Data represent means ± SEM of 6 independent experiments, *p < 0.05, Wilcoxon.

We then sought to confirm the relative abundance of candidates identified by our proteomic analysis. We evaluated the levels of the different adhesion proteins in both lEVs and their parental cells by Western blot. We found that shear stress did not affect the cellular levels of most of the candidates, except for the long isoform of MCAM, which was more abundant in HUVECs exposed to LSS conditions (Figure 4). However, PECAM‐1, MCAM (long isoform), as well as integrins α5, α6 and β1 were found to be enriched in LSS‐lEVs compared to HSS‐lEVs, thereby confirming the results obtained by quantitative proteomics.

**FIGURE 5:**
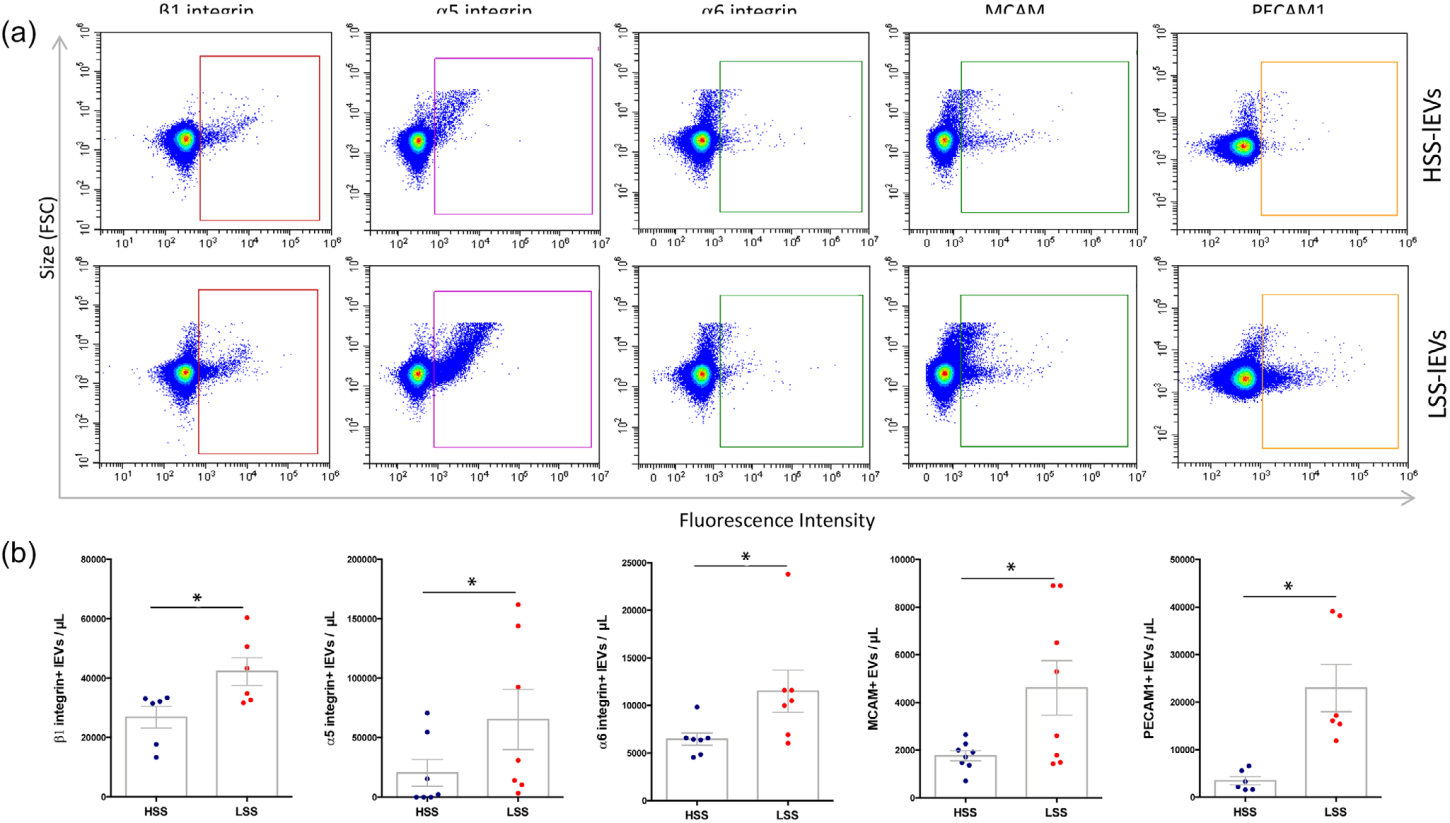
Adhesion protein are enriched at the surface of LSS‐lEVs. (a) Representative flow cytometry dot plots of HUVEC HSS‐lEVs (top panels) or LSS‐lEVs (bottom panels) labelled with antibodies against β1 integrin, α5 integrin, α6 integrin, MCAM or PECAM1. (b) Data represent means ± SEM of 6–8 independent experiments. *p < 0.05, Wilcoxon.

We next assessed the presence of the different adhesion proteins at the surface of lEVs by sensitive flow cytometry. lEVs produced by HUVECs exposed to HSS or LSS conditions were first stained with Membright to discern them from background noise. We then stained EV surfaces with antibodies directed against PECAM‐1, MCAM and integrins α5, α6 and β1. All five of these candidates were significantly enriched at the surface of LSS‐lEVs when compared to HSS‐lEVs (Figure 5). Triton detergent exposure (0,05%) decreased over 90% of the positive labelling (Figure S4).

### 3.5 LSS‐lEV uptake by HUVECs is in part mediated by PECAM‐1 and MCAM

**FIGURE 6:**
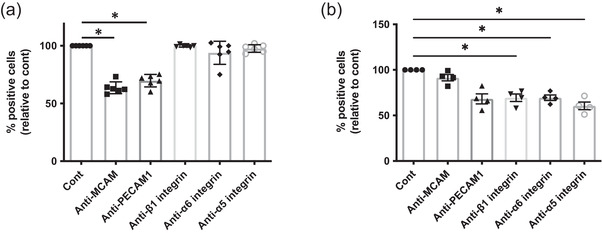
MCAM and PECAM1 are involved in endothelia EV uptake by HUVECs. Fluorescently‐labelled HUVEC‐derived LSS‐lEVs were pre‐incubated with neutralising antibodies targeting MCAM, PECAM1, β1 integrin, α5 integrin or α6 integrin. lEVs were then incubated for 90 min with HUVECs exposed to LSS conditions for 24 h (a) or THP1 cells (b). % of cells positive for EV signal, relative to control lEVs (Cont) was analysed by flow cytometry. Data represent means ± SEM of 4–6 independent experiments. *p < 0.05, Friedman test.

Having identified the several adhesion proteins enriched at the surface of LSS‐lEVs, we wanted to verify if they were functional and involved in the uptake process by endothelial cells. We assessed the potential role of PECAM‐1, MCAM and integrins α5, α6 and β1 using neutralising antibodies. Pre‐incubating lEVs with anti‐MCAM and anti‐PECAM‐1 antibodies resulted in a reduction of their uptake in a dose dependent manner (Figure S5A). At a concentration of 10 μg/mL, anti‐MCAM and anti‐PECAM‐1 neutralising antibodies blocked uptake by approximately 40% and 30% respectively, whilst blocking the different integrins had no impact on lEV uptake by endothelial cells (Figure 6a). Surprisingly, there was no additive effect of neutralising both PECAM1 and MCAM (Figure S5B). We also assessed the potential role of these adhesion proteins in the uptake of endothelial EVs by monocytic cells (THP‐1). Here, we found that blocking PECAM‐1 and integrins α5, α6 or β1 significantly reduced uptake, whilst blocking MCAM had no effect (Figure 6b). These data suggest that the surface expression of PECAM‐1 and MCAM is partially responsible for the uptake of LSS‐lEVs by HUVECs, whilst the presence of integrins may be useful for incorporation by other cell types such as monocytes.

### 3.6 Exposing endothelial cells to LSS increases the release of mitochondrial and endolysosomal material in lEVs

**FIGURE 7:**
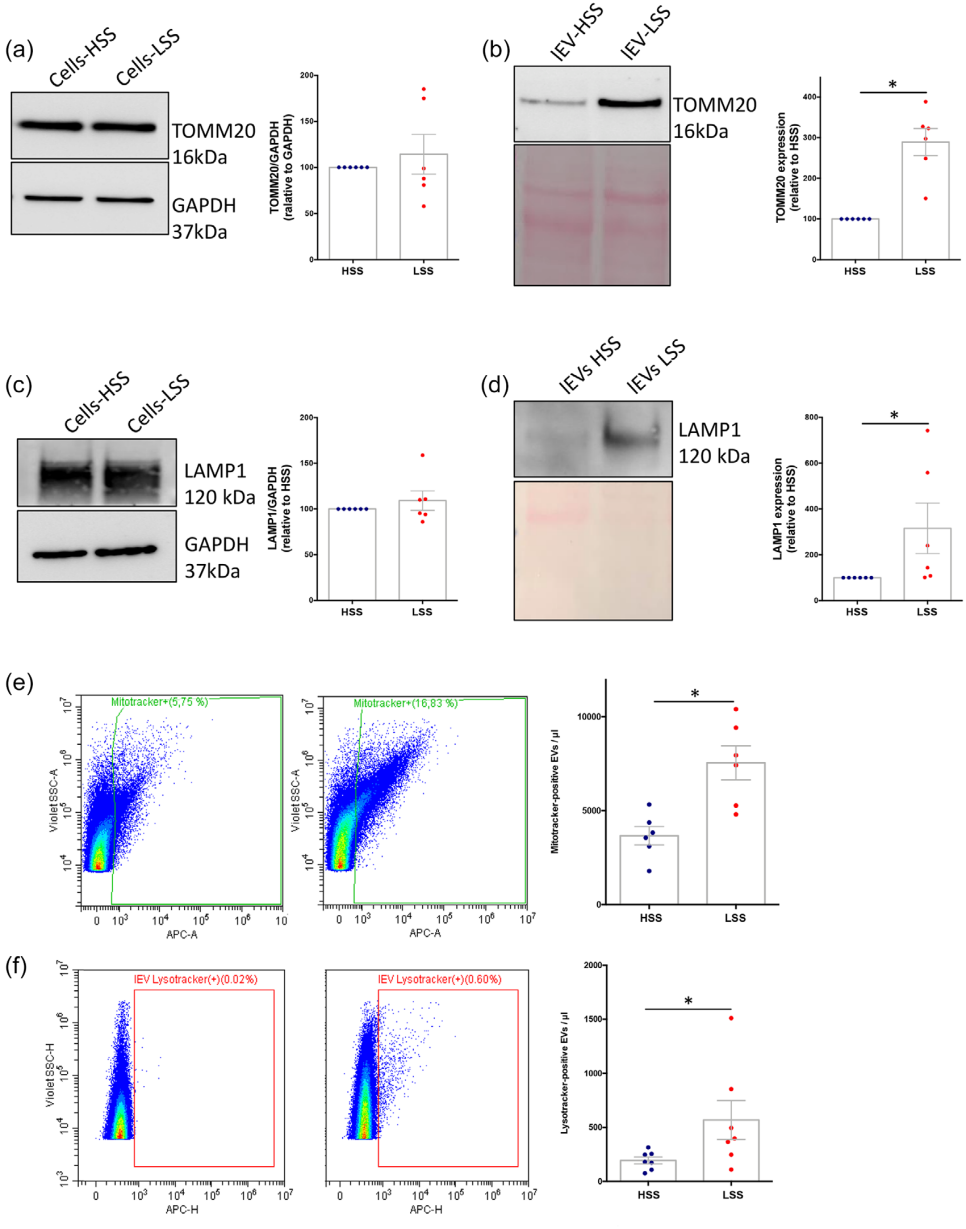
LSS‐lEVs are enriched in mitochondrial and endolysosomal material. Western blot analysis of TOMM20 (a and b) and LAMP1 (c and d) levels in HUVEC cellular (left panels) and lEV (right panels) protein lysates. HSS‐lEV and LSS‐lEV were stained with Mitotracker (e) or Lysotracker (f) and analysed by flow cytometry. Data represent means ± SEM of 6–7 independent experiments, *p < 0.05, Wilcoxon.

As our proteomic study had revealed an enrichment of mitochondrial and endolysosomal proteins in LSS‐lEVs, we sought to validate this by Western blot and sensitive flow cytometry. Whilst there was no difference in the cellular levels the mitochondrial protein Tomm20 or the endolysosomal protein LAMP1 (Figure 7a,c), we found higher levels of both markers in lEVs produced in LSS compared to HSS conditions (Figure 7b,d). Similarly, LSS‐lEV showed a higher staining for Mitotracker and Lysotracker (Figure 7e,f). These data suggest that endothelial cells exposed to LSS redirect mitochondrial and endolysosomal material towards a secretory pathway.

### 3.7 Uptake of LSS‐lEVs elevates ROS levels in endothelial cells

**FIGURE 8:**
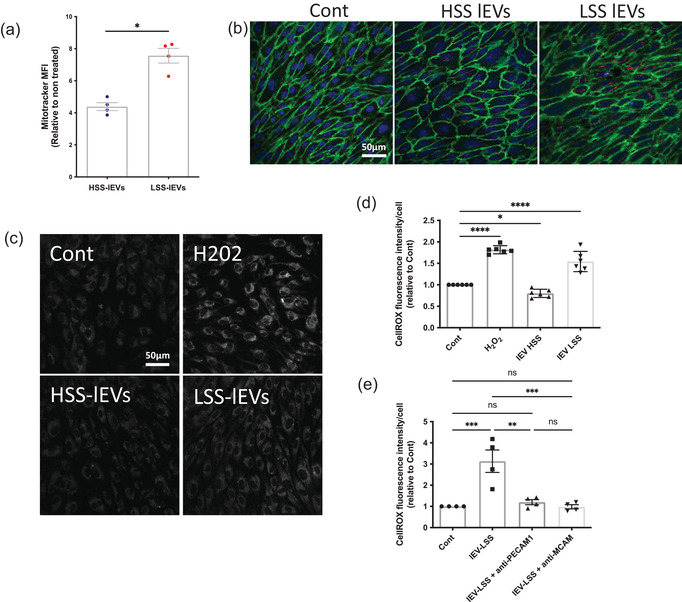
Effect of LSS‐lEVs on endothelial ROS levels. (a) LSS‐lEVs were pre‐stained with Mitotracker and deposited on HUVECs for 4 h. Mitotracker median fluorescent intensity per cell, relative to cells treated without EVs, was analysed by flow cytometry. Data represent means ± SEM of four independent experiments, *p < 0.05, Paired *t* test. (b) LSS‐lEVs were pre‐stained with Mitotracker and deposited on HUVECs for 4 h. Cells were then stained for Cadherin‐5 (green) and imaged by confocal microscopy. (c and d) HUVECs were incubated overnight with or without HSS‐lEVs or LSS‐lEVs. H_2_O_2_ was added in corresponding condition for the last hour of the experiment. Cells were stained with CellROX and immediately imaged. Fluorescent signal intensity was quantified from confocal images. Data represent means of fluorescence intensity per cell, relative to non‐treated cells (Cont), ± SEM of six independent experiments. (e) lEVs were pre‐incubated with or without neutralising antibodies against PECAM1 or MCAM. lEVs were then incubated overnight with HUVECs. Cells were stained with CellROX and immediately imaged. Fluorescent signal intensity was quantified from confocal images. Data represent means of fluorescence intensity per cell, relative to non‐treated cells (Cont), ± SEM of four independent experiments. *p < 0.05, **p < 0.01, ***p < 0.001, ****p < 0.0001, Friedman test.

We next wondered if the EV content could be transferred to recipient cells, and how that might affect their phenotype. Prestaining EVs with the Mitotracker dye allowed us to follow the transfer of mitochondrial material from EVs to cells. Cells exposed to LSS‐lEVs presented higher Mitotracker signal than those exposed to HSS‐lEVs, indicating higher incorporation of mitochondrial material (Figure 8a). Similarly, confocal imaging revealed higher staining in cells exposed to LSS‐lEVs (Figure 8b).

As damaged mitochondria are a known source of reactive oxygen species (ROS; Zorov et al., 2014), we investigated ROS levels in HUVECs exposed to lEVs produced under HSS or LSS conditions. We used a 30 min hydrogen peroxide treatment as a positive control and found that it almost doubled ROS levels in HUVECs. Overnight exposure of endothelial cells to HSS‐lEVs resulted in a 20% decrease in ROS levels. Conversely, exposing cells to LSS‐lEVs led to 50% increase in ROS levels (Figure 8c,d). Furthermore, exposing cells to HSS‐EVs or LSS‐EVs led to an increase in ICAM‐1, suggesting EV‐uptake had a pro‐inflammatory effect. There was no difference between cells exposed to HSS‐EVs and LSS‐EVs however. We also found that exposing cells to LSS‐lEVs resulted in an increase in levels of the senescence marker p21 (Figure S6).

Interestingly, the pro‐oxidative effects of LSS‐lEVs were completely blocked when EVs were pre‐incubated with PECAM1‐ or MCAM‐neutralising antibodies (Figure 8e). This indicates that the pro‐oxidative effects are caused by adhesion protein‐mediated attachment and/or endocytosis of EVs.

## 4 DISCUSSION

Over the past decade, EVs have emerged as crucial intercellular communication vehicles. By lining the vessel wall, endothelial cells are ideally positioned to be a significant contributor to circulating EVs. Endothelial‐EV composition and uptake is influenced by multiple factors including inflammation and thrombosis (Mathiesen et al., 2021). In this study, we demonstrate that shear stress is a key determinant in the way endothelial cells both produce and uptake EVs.

Our first major finding was that there was a higher concentration of EVs in the conditioned media of HUVECs cultured under HSS conditions compared to LSS conditions. As the number of EVs in the extracellular space is a balance between their rate of release and uptake, it is difficult at this point to conclude on the relative impact of each factor. Whilst we did not observe a difference in the average size of EVs produced under HSS or LSS conditions, we cannot exclude changes in size might be below detection levels. In this study, however we were able to show, in vitro and in vivo, that endothelial cells exposed to LSS conditions exhibited a higher rate of EV uptake than EVs exposed to HSS, mainly through a clathrin‐dependent pathway. This was observed for EVs of different cellular origin, including platelets, red blood cells, or peripheral blood mononuclear cells, but the most robust uptake was observed for endothelial EVs released under low shear stress (LSS‐lEVs). Altogether, the present data support the hypothesis that endothelial EVs present in the blood can be re‐incorporated by endothelial cells of the arteries. Using imaging flow cytometry, Banizs et al. (2018) had also observed that EV uptake by mouse aortic endothelial cells was essentially a receptor‐mediated clathrin‐dependent process. Higher endocytic activity under low or oscillatory shear stress does not seem to be restricted to endothelial EVs however, as it has also been described for erythrocyte derived EVs, as well as polystyrene or gold nanoparticles (Charwat et al., 2018; Qin et al., 2021). This suggests that the uptake of various types of particles is enhanced by low magnitude shear stress, and implies that atheroprone regions of the arterial tree may be targetable by therapeutic nanovesicles. Whilst the underlying mechanisms remain to be elucidated, Qin et al. suggest that LSS‐induced oxidative stress may be implicated, as oxidative stress genes are expressed earlier than pro‐inflammatory genes in response to LSS, and antioxidant treatment reduced EV uptake (Ajami et al., 2017; de Keulenaer et al., 1998; Hsiai et al., 2007; Hsieh et al., 2014).

The uptake mechanism involves protein interactions which facilitate EV retention and subsequent endocytosis. Our data showed that LSS‐lEVs were more adherent than HSS‐lEVs, we therefore sought to identify adhesion proteins that could mediate these effects. Using proteomic analysis, combined with Western blot and sensitive flow cytometry, we were able to uncover several adhesion proteins that are enriched at the surface of LSS‐lEVs. Since most of these proteins showed no significant differences in cellular proteins levels, we hypothesise that they were selectively directed towards a secretory pathway in LSS conditions. As key players in cellular adhesion, several integrins have been shown to be crucial in cell‐EV interactions (Deregibus et al., 2007; Esmaeili et al., 2022; Mulcahy et al., 2014; Rana et al., 2012). Here we found that integrins α5, α6 and β1 were enriched at the surface of LSS‐lEVs. Though they were involved in endothelial EV uptake by monocytes, we found that they played little role in uptake by endothelial cells. PECAM1 and MCAM, on the other hand, were required for EV uptake by endothelial cells. Whilst MCAM had previously been implicated in the interaction of melanoma cell‐derived EVs with endothelial cells (Ghoroghi et al., 2021), this represents, to our knowledge, the first description of an involvement of PECAM1 in EV uptake by endothelial cells. Interestingly, there was no additive effect of neutralising both MCAM and PECAM1, suggesting that they may be located on the same subset of EVs, and may work in tandem to allow EV endocytosis. Together, these data reinforce the idea that EV internalization is a selective process. Surface proteins seem to serve as molecular barcodes, targeting EVs towards specific recipient cells, and therefore playing a role in the biodistribution of circulating EVs.

Several studies have shown evidence for the presence of extracellular mitochondria, either freely (al Amir Dache et al., 2020; Joshi et al., 2019; Puhm et al., 2019) or encapsulated within EVs (D'Acunzo et al., 2021; Todkar et al., 2021; Torralba et al., 2016). Although the intracellular mechanisms regulating this process are still emerging, evidence points towards the involvement of mitochondria‐derived vesicles. These are small vesicles which deliver damaged mitochondrial content to late endosomes/lysosomes in a Pink1/Parkin‐dependent manner (Sugiura et al., 2014). Remarkably, autophagy‐deficient cells displayed higher extracellular mitochondria release, implying that perturbation of the mitophagy pathway prompts mitochondria expulsion (Choong et al., 2021). Thus, this process appears to be a comparable yet distinct quality control pathway from conventional mitophagy. It might be involved in ensuring mitochondrial homeostasis in the event of mild mitochondrial damage, such as oxidative stress, whilst mitophagy is triggered by acute mitochondrial depolarization (Amari & Germain, 2021). Our proteomic analysis of endothelial EVs revealed an enrichment of endolysosomal and mitochondrial proteins in LSS‐lEVs. As we had previously demonstrated that the autophagic flux is hindered in LSS conditions (Vion et al., 2017), we hypothesise that endothelial cells respond by packaging damaged mitochondria for secretion in EVs.

Our data indicate that this mitochondrial material can be transferred to new endothelial cells via EVs, and may be involved in elevating ROS levels in recipient cells. This pro‐oxidative effect was blocked by neutralising antibodies targeting PECAM1 or MCAM, which suggests that it is mediated by EV uptake. As mentioned above, endothelial cells exposed to LSS seem to have increased endocytic activity but reduced degradative capacity. Together, these processes may lead to accumulation of noxious pro‐oxidative material which contributes to sensitising cells to inflammatory stimuli in atheroprone regions (Puhm et al., 2019; Zorov et al., 2014). Interestingly, exposure to HSS‐lEVs had a small antioxidant effect, even though these EVs were barely taken up. Notably, we were able to detect several components of the anti‐oxidant machinery in HSS‐lEVs, including Glutathione S transferase, Peroxiredoxin‐1/2/6 and Glutamate dehydrogenase 1 (Table S1). Whilst their eventual implication in these effects remains to be determined, previous work has shown a transfer of the anti‐oxidant machinery via EVs (Bodega et al., 2017, 2018). It is therefore tempting to imagine leveraging the knowledge obtained in this study to target therapeutic material to endothelial cells in areas of LSS. In that sense, engineering HSS‐EVs to overexpress MCAM and/or PECAM1 could bolster their uptake and anti‐oxidant effects on recipient cells. These EVs could therefore promote an atheroprotective endothelial phenotype.

### 4.1 Study limitations

First, due to the lack of commercially available arterial endothelial cell lines which adequately respond to shear stress, we chose to perform our experiments with a well characterised model of venous endothelial cells, available at very low passage number, and therefore retaining a physiological response to shear stress. Though venous and arterial endothelial cells share many genetic pathways, these two populations remain distinct. We should therefore be cautious when attempting to link the findings of this study to pathologies which mainly affect the aortic endothelium. Second, visualising EV release and uptake by cells exposed to flow in real‐time remains technically challenging. Whilst our results allow us to infer how shear stress impacts these processes, we lack a temporal analysis. Finally, we have focused our study on the protein cargo of EVs, specifically adhesion proteins, it would be interesting to examine how the nucleic acid and lipid content of EVs is affected by shear stress, whether they are transferred between endothelial cells and what consequences this would have on the cell's phenotype.

In conclusion, endothelial cells exposed to LSS, and therefore prone to developing atherosclerotic plaques, are characterised by higher incorporation of circulating endothelial EVs than those exposed to HSS. LSS endothelial cells also release EVs enriched in adhesion proteins and mitochondrial material, which may transfer noxious pro‐oxidative cargos to recipient cells.

## AUTHOR CONTRIBUTIONS

Pierre‐Michaël Coly and Shruti Chatterjee designed, performed, and analysed most of the experiments with the help of Christelle El Jekmek, Cécile Devue, Thomas Nipoti and Stephane Mazlan. Fariza Mezine performed and analysed EV flow cytometry. Maribel Lara Corona performed and analysed electron microscopy. Florent Dingli carried out the MS experimental work and Damarys Loew supervised MS and data analysis. Pierre‐Michaël Coly, Chantal M. Boulanger, Xavier Loyer and Guillaume van Niel wrote/reviewed the manuscript. Chantal M. Boulanger conceived and supervised the study.

## CONFLICT OF INTEREST STATEMENT

The authors report no conflict of interest.

## Supporting information

Supplementary Information

Supplementary Information

## Data Availability

The authors declare that the data supporting the findings of this study are available within the paper and its supplementary information files.
