# Supplementary material for: Low fluid shear stress stimulates the uptake of noxious endothelial extracellular vesicles via MCAM and PECAM‐1 cell adhesion molecules
Source: J Extracell Vesicles. 2024 Oct 14;13(10):e12414. doi: 10.1002/jev2.12414 (PMC11472237; doi:10.1002/jev2.12414)
Supplement: Supplementary file 1 — Supplementary Information [file JEV2-13-e12414-s002.docx]

**Supplementary Figures**


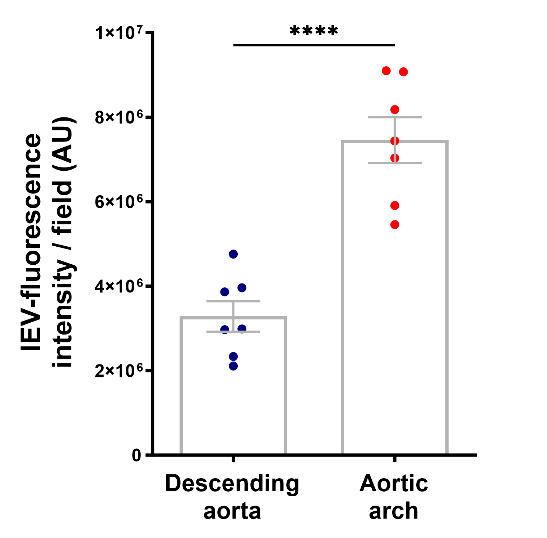


**Supplementary Figure 1:** Fluorescently-labeled SVEC4-10 LSS-lEVs were injected into the mouse bloodstream. Animals were sacrificed after 30 min and aortas were harvested, fixed and endothelial cells were labeled (Cadherin-5). The descending aorta and aortic arc regions were imaged by confocal microscopy (10 fields per condition). Data represent means ± SEM, N=7. ****P<0.0001; Paired t test.

**Supplementary Figure 2:** Gene Ontology, cellular component terms associated with proteins identified in endothelial EVs.

**Supplementary Figure 3**: HUVECs were exposed to HSS or LSS conditions for 24 h. Cells were then washed, detached, labeled with the Viability Dye eFluor™ 780 and analyzed by flow cytometry. Data are expressed as mean +/- SEM.


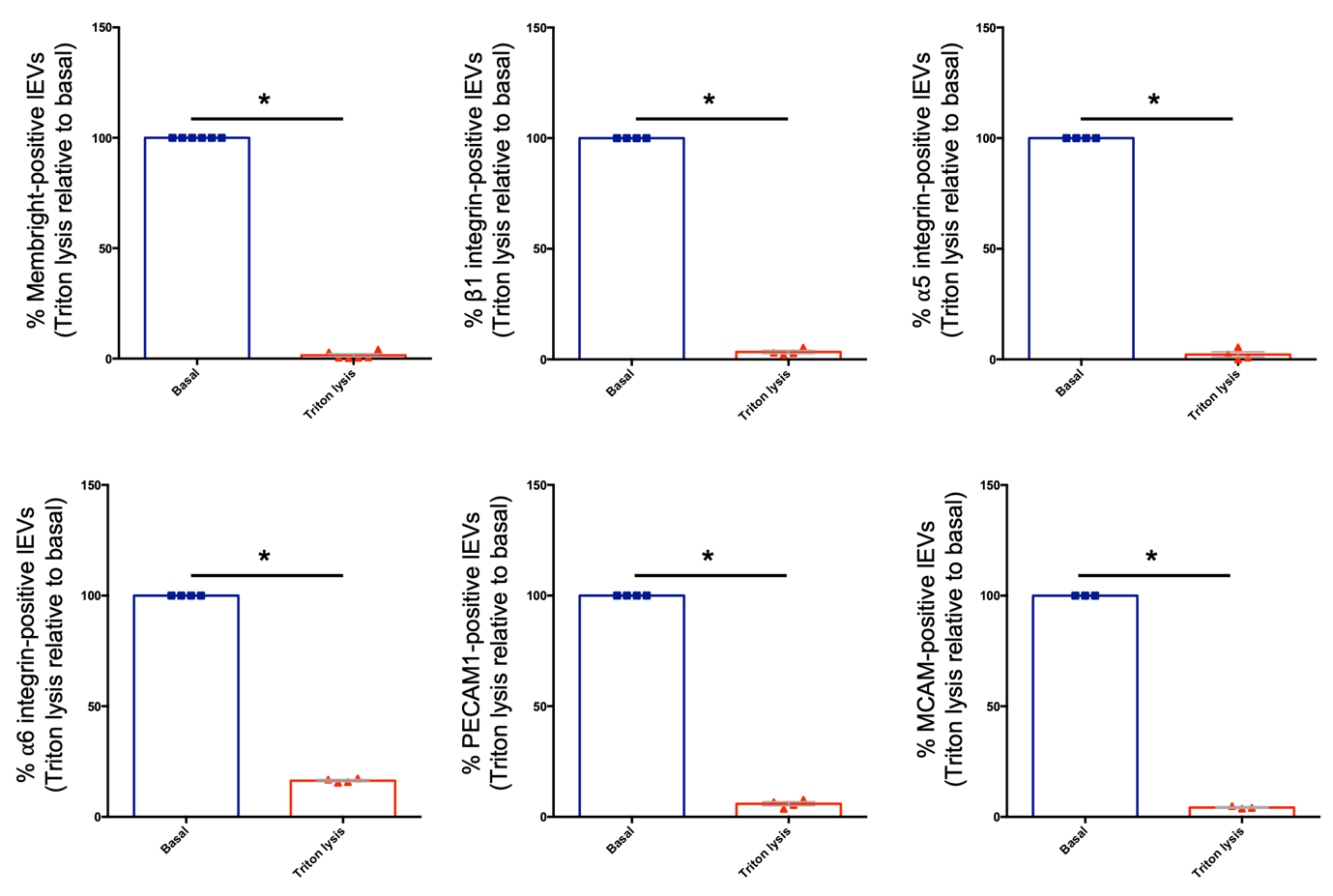


**Supplementary Figure 4**: Effect of Triton lysis on lEVs flow cytometry analysis. **(A)** Levels of lEVs positive for the different markers in absence or presence of Triton. Data are expressed as mean +/- SEM. *P < 0.05, paired t test.


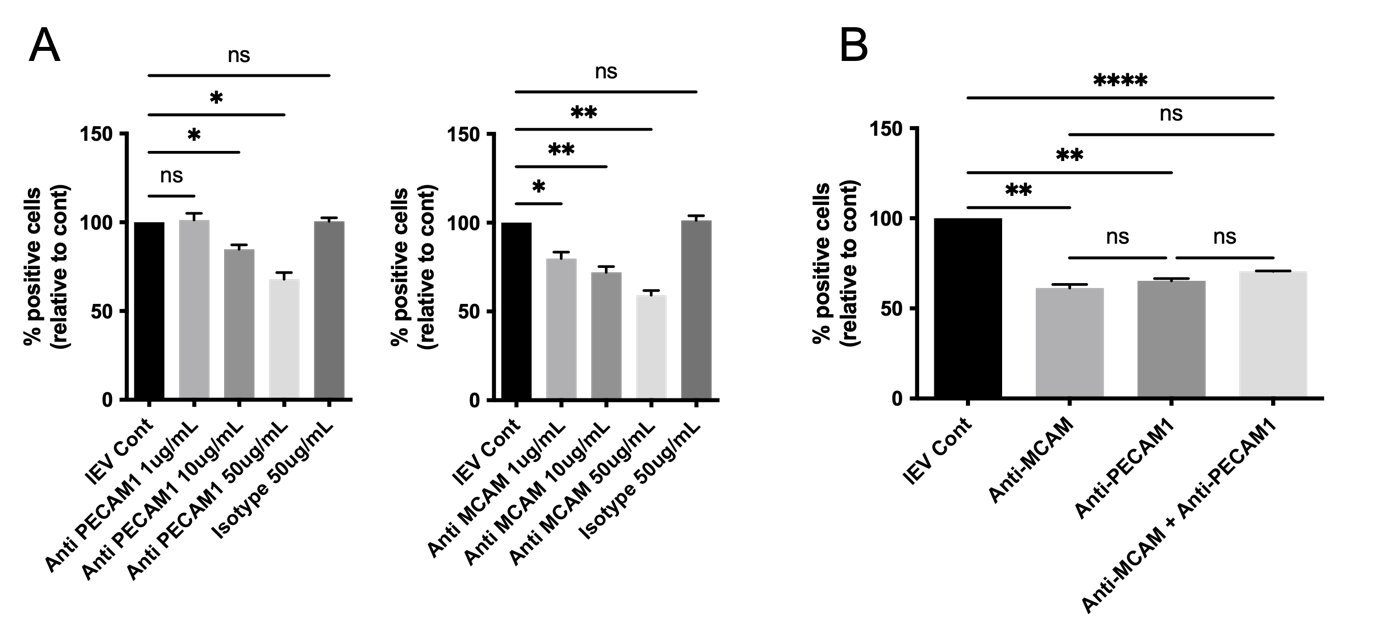


**Supplementary Figure 5:** Fluorescently-labeled HUVEC-derived LSS-lEVs were pre-incubated with different concentrations of either MCAM- or PECAM1-neutralizing antibodies **(A)** or with a combination of both **(B)**. lEVs were then incubated for 90 min with HUVECs. % of cells positive for EV signal, relative to control lEVs (Cont) was analyzed by flow cytometry. Data represent means ± SEM of 6-8 independent experiments. *P < 0.05, **P<0.01, ****P<0.0001; Friedman test.


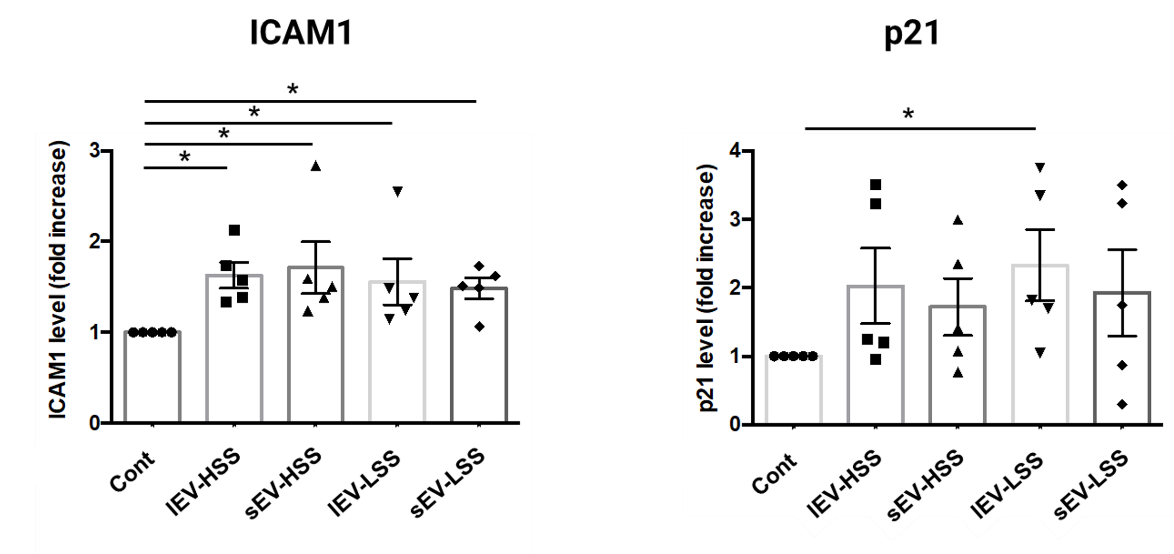


**Supplementary Figure 6:** HUVECs were incubated overnight with or without lEVs or sEVs produced under HSS or LSS conditions. Cells were then washed, lysed, and the levels of ICAM-1 (left panel) and p21 (right panel) were analyzed by Western blot. Data represent means, relative to non-treated cells (Cont), ± SEM of 5 independent experiments. *P<0.05, **P<0.01, ***P<0.001, ****P<0.0001, Friedman test.
